# Excitability of Rat Superficial Dorsal Horn Neurons Following a Neonatal Immune Challenge

**DOI:** 10.3389/fneur.2018.00743

**Published:** 2018-09-07

**Authors:** Melissa A. Tadros, Ihssane Zouikr, Deborah M. Hodgson, Robert J. Callister

**Affiliations:** ^1^Faculty of Health and Hunter Medical Research Institute, School of Biomedical Sciences and Pharmacy, University of Newcastle, Callaghan, NSW, Australia; ^2^Laboratory for Molecular Mechanisms of Thalamus Development, RIKEN, Wako, Saitama, Japan; ^3^Laboratory of Neuroimmunology, School of Psychology, University of Newcastle, Callaghan, NSW, Australia

**Keywords:** action potential, LPS, pain, potassium current, EPSC

## Abstract

Previous studies have shown that neonatal exposure to a mild inflammatory challenge, such as lipopolysaccharide (LPS, *Salmonella enteriditis*) results in altered pain behaviors later in life. To further characterize the impact of a neonatal immune challenge on pain processing, we examined the excitability of superficial dorsal horn (SDH) neurons following neonatal LPS exposure and subsequent responses to noxious stimulation at three time-points during early postnatal development. Wistar rats were injected with LPS (0.05 mg/kg i.p.) or saline on postnatal days (PNDs) 3 and 5, and later subjected to the formalin test at PNDs 7, 13, and 22. One hour after formalin injection into the plantar hindpaw, animals were euthanized (Ketamine, 100 mg/kg i.p.) and transverse slices from the lumbosacral spinal cord were prepared. Whole-cell patch-clamp recordings were made from SDH neurons (KCH_3_SO_4_-based internal, 22–24°C) on the ipsi- and contralateral sides of the spinal cord. Depolarising current steps were injected into SDH neurons to categorize action potential (AP) discharge. In both saline- and LPS-treated rats we observed age-related increases the percentage of neurons exhibiting tonic-firing, with concurrent decreases in single-spiking, between PND 7 and 22. In contrast, neonatal exposure to LPS failed to alter the proportions of AP discharge patterns at any age examined. We also assessed the subthreshold currents that determine AP discharge in SDH neurons. The rapid outward potassium current, I_Ar_ decreased in prevalence with age, but was susceptible to neonatal LPS exposure. Peak I_Ar_ current amplitude was greater in ipsilateral vs. contralateral SDH neurons from LPS-treated rats. Spontaneous excitatory synaptic currents (sEPSCs) were recorded to assess network excitability. Age-related increases were observed in sEPSC frequency and time course, but not peak amplitude, in both saline- and LPS-treated rats. Furthermore, sEPSC frequency was higher in ipsilateral vs. contralateral SDH neurons in LPS-treated animals. Taken together, these data suggest a neonatal immune challenge does not markedly affect the intrinsic properties of SDH neurons, however, it can increase the excitability of local spinal cord networks via altering the properties of rapid A-type currents and excitatory synaptic connections. These changes, made in neurons within spinal cord pain circuits, have the capacity to alter nociceptive signaling in the ascending pain pathway.

## Introduction

The long-term consequences of early life events, such as neonatal stress and infection, are well documented. For example, rats exposed to the bacterial mimetic, lipopolysaccharide (LPS), during the first postnatal week exhibit potentiated stress responses when tested as adults. This has been attributed to permanent alterations within the hypothalamic-pituitary-adrenal (HPA) axis (1). One commonly used method to reliably and rapidly activate the HPA axis is the formalin test. This test, initially described by Dubuisson and Dennis (2), involves formalin injection into the hindpaw. It causes an inflammatory response and characteristic bi-phasic pain-induced behaviors (flinching and licking). Our group has shown previously that rats exposed to LPS at postnatal days (PNDs) 3 and 5 exhibit increased formalin-induced behavioral responses during both pre-adolescence (PND 13; (3) and adulthood (PND 80–97; (4). These findings demonstrate that neonatal exposure to LPS can have long-lasting effects on pain behavior. Surprisingly, limited data exists on the spinal cord neurons and mechanisms that underlie these long-lasting changes in pain processing.

The superficial dorsal horn (SDH) of the spinal cord is the first processing node in the ascending pain pathway. We have known for more than two decades that peripheral immune challenges can increase the concentration of pro-inflammatory substances within the adult spinal cord (5). Moreover, bath application of pro-inflammatory substances to spinal cord slices can alter the properties of SDH neurons. These alterations include decreases in input resistance in response to prostaglandin E2 (6) and increased excitatory drive following exposure to BDNF (7). In addition, formalin injection in adult animals can increase spinal cord metabolic activity (8) and alter dorsal horn signaling (9, 10). Thus, it is clear that an early immune challenge causes changes in the adult spinal cord, however, little is known about the nature of these changes or when they occur during the early postnatal period. This is important as plasticity within the rodent spinal cord occurs during critical periods during postnatal development (11).

In order to understand the neuronal mechanisms underlying the long-term changes in adult pain behaviors after neonatal insults, several studies have investigated SDH neuron properties in response to neonatal injuries. Injection of inflammatory agents into the hindpaw of neonatal rats results in spreading of the central arbors of primary afferent fibers, 8–10 weeks later (12). This has implications for overall network excitability. Indeed, when examining network excitability following a surgical incision of the mid-thigh, Li, and colleagues (13) observed an increase in the frequency of excitatory postsynaptic currents (EPSCs) within SDH neurons. Further studies utilizing a similar neonatal surgical incision also revealed alterations in the intrinsic properties of individual SDH neurons in the adult (14). Together, these studies indicate that the SDH contains a neuronal population that is highly plastic during the early postnatal period and susceptible to perturbations after peripheral inflammation and/or injury.

Despite the evidence that neonatal injury, immune challenge and formalin injection can alter SDH properties in adult rats, limited information exists on the effect of neonatal exposure to LPS and subsequent formalin injection on the developmental trajectory of SDH neurons. Previously, our group has examined the intrinsic properties of SDH neurons and observed altered intrinsic properties of LPS-challenged rats at PND 22 formalin challenge (3). Based on these findings we proposed that a neonatal exposure to LPS can alter the excitability of SDH neurons long after the initial immune response has passed. Therefore, in order to build upon these previous initial findings, the present study reports whole-cell responses from SDH neurons following formalin injection in rats previously challenged with a neonatal exposure to LPS or saline. We demonstrate significant developmental changes in rat SDH neurons and an increase in overall network excitability within the SDH following neonatal inflammation. These data provide insight into the mechanisms underlying neuron plasticity at the first node in the ascending pain pathway during a noxious event following an early life immune challenge.

## Materials and methods

### Experimental procedures

All protocols were approved by the University of Newcastle Animal Care and Ethics Committee, and were undertaken in accordance with the 2004 National Health and Medical Research Council of Australia Code of Practice covering the use of animals for scientific purposes. Eight naïve female Wistar rats were obtained from the University of Newcastle Animal House and allowed 1-week acclimatization prior to mating in a vivarium. Mating resulted in 86 offspring, and 58 were used in this study. After birth, at PNDs 3 and 5, the rat pups were briefly removed from their home boxes, weighed and given an IP injection of either LPS (*Salmonella enterica*, serotype *enteriditis*; Sigma Aldrich chemical Co., USA, dissolved in sterile pyrogen-free saline, 0.05 mg/kg) or an equal volume of saline (Livingstone International, Australia). We have previously confirmed that this concentration of LPS raises the circulating levels of cytokines (IL-1β) and plasma corticosterone (3), as well as the levels of pro-inflammatory cytokines IL-1β and TNF-α in the hippocampus (15). The pups were then left undisturbed with their dams in a temperature- (21 ± 1°C) and humidity- (60%) controlled environment, under a 12/12 h light/dark cycle with food and water available *ad libitum*. All pups from the same litter were treated identically.

### Formalin behavioral testing and analysis

Rats were randomly assigned to three age groups (PND 7, 13, and 22) with a maximum of three pups per litter being assigned to each group. On the test-day, each rat received a 10 μl injection of formalin into the plantar surface of the hindpaw via a 31 gauge needle; PND 7 rats were injected with 0.5% formalin, PND 13 with 0.8% formalin, and PND 22 with 1.1% formalin. These formalin concentrations were used because previous studies demonstrated they are sufficient to induce the characteristic bi-phasic behavioral response at each age (16, 17). The test apparatus and conditions have been described elsewhere in detail by our group (17). We did not examine any responses to injection of saline into the hindpaw as it has been previously shown that rats subjected to such an injection do not display flinching or licking behaviors when tested at any developmental stage (16, 18, 19).

Behavioral responses to the formalin injection were scored according to the method of Wheeler-Aceto and Cowan (20). Testing in the hour following formalin injection was divided into an early (the first 5 min) and late phase (10–60 min) during which flinching frequency and licking duration of the injected paw (in seconds) were scored. A detailed description of the statistical analysis and resulting data can be found in a previous publication from our group (3). Briefly, whilst no differences in behavioral responses were observed at PND 7, an increase in licking was observed at PND 13 and an increase in flinching behaviors at PND 22.

### Electrophysiological recordings

Following the formalin test, spinal cords from 58 rats (PND 7 = 15; PND 13 = 18; PND 22 = 25; equally distributed between treatment groups and sexes) were dissected for subsequent electrophysiological recordings from superficial dorsal horn (SDH, laminae I, and II) neurons. For this procedure the rats were anesthetized with Ketamine (100 mg/kg) and decapitated. The vertebral column and posterior thoracic wall were isolated and rapidly immersed in ice cold sucrose substituted artificial cerebrospinal fluid (sACSF) containing (in mM): 250 sucrose, 25 NaHCO_3_, 10 glucose, 2.5 KCl, 1 NaH_2_PO_4_, 1 MgCl_2_, and 2.5 CaCl_2_, continuously bubbled with 95% O_2_- 5% CO_2_ (pH of 7.3–7.4; (21). The lumbosacral enlargement of the spinal cord was then removed and marked to allow identification of the ipsi- and contralateral dorsal horns - relative to the injection site. Transverse slices from L3-5 spinal segments (300 μm thick) were prepared using a vibratome (Leica VT1200s, Leica Microsystems, Wetzlar, Germany). Slices were transferred to an interface storage chamber containing ACSF (118 mM NaCl substituted for sucrose in sACSF) and allowed to equilibrate for 1 h at room temperature (22–24°C) before recording commenced.

Slices were transferred to a recording chamber (volume 0.4 ml) and continually superfused with ACSF (4–6 bath volumes/minute). Recording temperature was maintained at 22–24°C via an in-line temperature control unit (TC-324B, Warner Instruments, Hamden, CT). Whole-cell patch-clamp recordings were obtained from SDH neurons visualized with infrared differential contrast optics and an infrared-sensitive camera (Rolera-XR, Olympus, NJ). Patch pipettes (3–4 MΩ resistance) were prepared from thin walled borosilicate glass (PG150T-15, Harvard Apparatus, Kent UK) and filled with a potassium-based internal containing (in mM): 135 KCH_3_SO_4_, 6 NaCl, 2 MgCl_2_, 10 HEPES, 0.1 EGTA, 2 MgATP, 0.3 NaGTP, pH 7.3 (with 1 M KOH). Whole-cell patch-clamp recordings were obtained using a Multiclamp 700B Amplifier (Molecular Devices, Sunnyvale, CA). The whole-cell recording configuration was first established in voltage clamp mode (holding potential −60 mV). Series resistance was measured from the averaged response (five trials) to a hyperpolarizing pulse (5 mV amplitude, 10 ms duration). This was measured at the beginning and end of each recording session and data were rejected if values changed by >20%. While in the voltage clamp recording mode, the presence of the major subthreshold currents, known to be present in rodent SDH neurons (21, 22), was assessed by delivering a hyperpolarizing pulse (to −90 mV, 1 s duration) immediately followed by a depolarizing step (to −40 mV, 200 ms duration). This was repeated five times to obtain an average for analysis. In addition to the above protocol, spontaneous excitatory postsynaptic events (sEPSCs) were recorded in voltage clamp over a period of 60–120 s from a holding potential of −70 mV.

Following the completion of the above voltage clamp protocols, the amplifier was switched to current clamp mode. The membrane potential observed ~15 s after this switch was taken as resting membrane potential (RMP). All current clamp recordings were made from this potential and reported membrane potentials have been corrected for a 10 mV liquid junction potential (23). Individual action potential (AP) properties and discharge categories were examined by injecting a series of depolarizing and hyperpolarizing current steps (20 pA increments, 800 ms duration, delivered every 8 s).

### Data capture and analysis

Data were digitalized online (sampled at 20 kHz; sEPSCs filtered at 2 kHz, all other data filtered at 6 kHz) via an ITC-16 computer interface (Instrutech, Long Island, NY) and stored on a Macintosh computer using Axograph X software (Molecular Devices). Data were analyzed offline using Axograph X software. sEPSCs were detected and captured using the scaled template method (24, 25). Upon inspection, events were rejected if they overlapped or did not include a stable baseline prior to the rising phase of the captured event. Several sEPSC parameters were measured, including peak amplitude and rise and decay times as well as the charge transfer (26). Because the rapid A-type potassium current (I_Ar_) was the dominant current observed at all ages in both saline- and LPS-treated rats, its features were analyzed further as done previously (21). I_Ar_ amplitude was measured by subtracting the amplitude of any steady-state current (in the last 50 ms of the depolarizing step) from the maximal I_Ar_ current peak. The decay phase of the I_Ar_ response was fit with a single exponential (over 20–80% of its falling phase). Individual APs were captured using the derivative threshold method, using the optimum threshold for each age group examined (PND 7, *dv/dt* = 10; PND 13, *dv/dt* = 15; PND 22, *dv/dt* = 20). AP threshold was measured at the inflection point during the rising phase of the AP. Rheobase current was defined as the smallest step-current that elicited at least one AP. The amplitude of each AP was measured as the difference between its threshold and maximum positive peak. AP half-width was calculated at 50% of AP amplitude. AP afterhyperpolarization was measured as the difference between AP threshold and its maximum negative peak.

### Statistics

Comparisons were made in one of two ways: (1) across postnatal developmental, by comparing data for contralateral SDH neurons at each age within each treatment group, or (2) to assess the impact of neonatal exposure to LPS upon a subsequent injection of formalin, by comparing treatment groups within each age group using SPSS v25. G tests, with Williams' correction, were used to determine whether the proportions of discharge categories, responses to hyperpolarizing current and subthreshold currents differed between either ages or treatment groups. Properties of the rapid A current and sEPSC parameters were analyzed by three-way ANOVAs with Tukey *post-hoc* tests. Data that failed Levene's test for homogeneity of variance were compared using the non-parametric Mann-Whitney test. Statistical significance was set at *p* < 0.05 and all data are presented as means ± SEM.

## Results

A total of 228 recordings (PND 7 = 65; PND 13 = 77; PND 22 = 86) were obtained from SDH neurons in 58 rats (PND 7 = 15; PND 13 = 18; PND 22 = 25; equally distributed between treatment groups and sexes). Recordings were made from neurons classified as either ipsi- or contralateral according to injection side (i.e., left or right hindpaw), and sample numbers reflect those from previous studies examining SDH neuronal properties (11, 21). Recording conditions, as assessed by series resistance and holding current, did not differ between any of the groups examined in this study. Data were compared in two ways: (1) during postnatal development—by comparing contralateral SDH neuron responses across the three ages examined, or (2) to assess the impact of neonatal exposure to LPS upon a subsequent injection of formalin—by comparing ipsi- or contralateral SDH neuron responses from saline- or LPS-treated rats within each age group. In our previous study, we reported the intrinsic properties of these neurons, including input resistance, resting membrane potential, rheobase and AP properties (3). Previously, we reported remarkably similar intrinsic properties at PND 7 and 13, with a decrease in input resistance and AP amplitude in ipsilateral SDH neurons from LPS-treated animals at PND 22. These previous data underpin our current hypothesis that a neonatal exposure to LPS alters the excitability of SDH neurons long after the initial immune response has passed. To extend our previous findings, in the current study we provide a detailed account of the properties that shape the excitability of these SDH neurons within dorsal horn circuits. These properties include the excitability of individual SDH neurons as measured by injection of current steps, the whole-cell currents underlying neuronal excitability, and the excitability of the SDH network by measuring the spontaneous excitatory post-synaptic currents (sEPSCs).

### Action potential discharge

**Figure 1:**
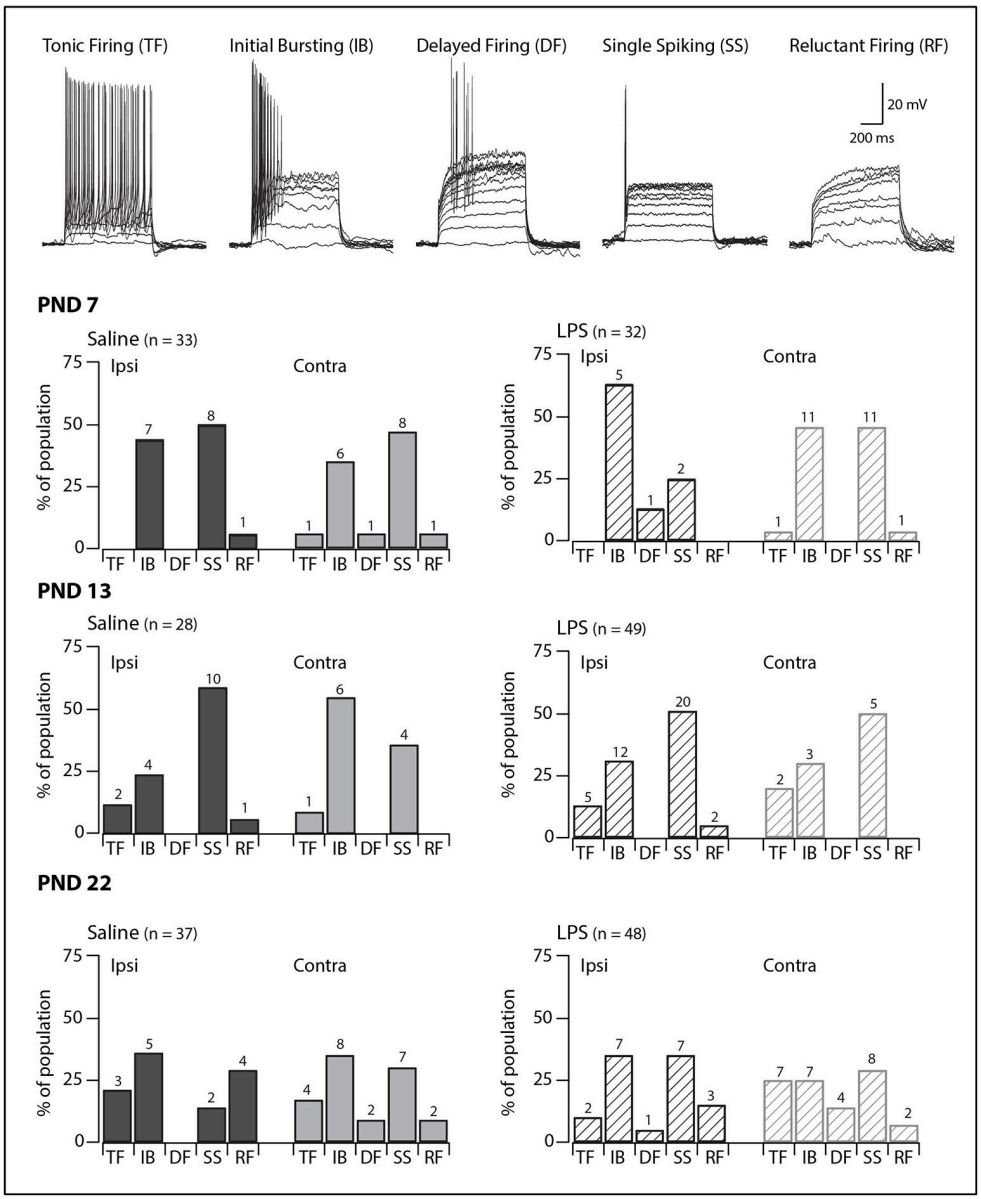
Prevalence of AP discharge categories. Upper traces: representative traces displaying the five types of AP discharge observed in SDH neurons. All traces are from saline-treated PND 22 SDH neurons (holding potential −60 mV). Lower bar plots: demonstrate the prevalence of AP discharge categories at the ages examined (PND 7, 13 and 22, *n* values for each bar indicated). SDH neurons were recorded both ipsilateral (dark gray) and contralateral (light gray) to the formalin injection side in animals pre-treated with either saline (solid bars) or LPS (hashed bars) when they were neonates. In both treatment groups, an age-related increase in tonic and delayed firing neurons with a concurrent decrease in single spikers was observed. However, there were no significant differences in the proportions of the discharge categories between saline- and LPS-treated, or between the ipsi- and contralateral SDHs in any of the three age groups.

SDH neurons displayed one of five classic AP discharge patterns, at resting membrane potential, in response to depolarizing current step injection (Figure 1, upper panel). Tonic firing neurons discharged APs for the entire duration of the current injection; initial bursting neurons discharged a brief burst of APs at the onset of current injection; delayed firing neurons discharged APs after a delay from current onset; single spiking neurons discharged only a single AP at the onset of current injection regardless of current step magnitude; and reluctant firing neurons did not discharge any APs in response to depolarizing current injection. Reluctant firing neurons were included in this dataset as they had similar input resistance and RMP to the other categories but were capable of AP discharge under alternative recording conditions, such as when membrane potential was altered (27, 28).

We observed age-related changes in the proportions of AP discharge categories. These changes were observed in both saline- and LPS-treated rats (*G statistic* = 10.14, *p* = 0.04 and *G statistic* = 12.01, *p* = 0.02, respectively). The proportions of tonic firing neurons increased, whereas that of single spiking neurons decreased (Figure 1, lower panels). In contrast, there were no differences in the proportions of the various AP discharge categories between saline- and LPS- treated animals at any of the three ages examined. There were also no differences in the prevalence of the AP discharge categories in neurons from the ipsi- and contralateral sides of the spinal cord. Taken together, these data show rat SDH neurons are still plastic (i.e., altering their properties or maturing) during the first 3 weeks of postnatal development, however, the development of AP discharge categories is unaffected by neonatal exposure to LPS.

### Responses to hyperpolarizing current injection

**Figure 2:**
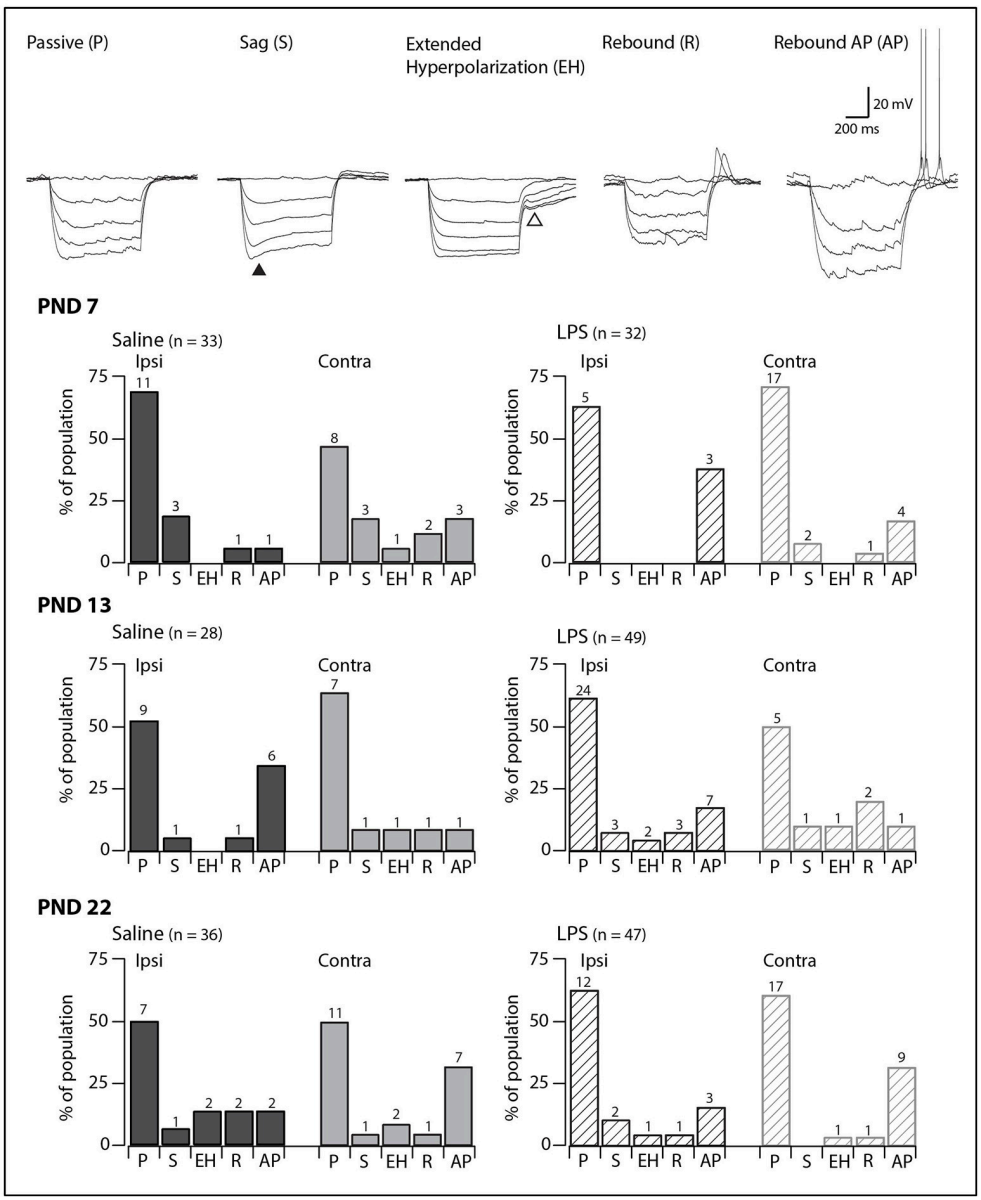
Prevalence of responses to hyperpolarizing current injection. Upper traces: representative traces showing the five types of responses to hyperpolarizing current injection observed in SDH neurons. All traces are from saline-treated PND 22 SDH neurons (holding potential −60 mV). Closed triangle indicates the “sag” observed during hyperpolarizing current injection. Open triangle indicates the extended hyperpolarization observed upon release from hyperpolarization. Lower bar plots: demonstrate the prevalence of responses to hyperpolarizing current injection at the ages examined (PND 7, 13, and 22, *n* values for each bar indicated). SDH neurons were recorded both ipsilateral (dark gray) and contralateral (light gray) to formalin injection in animals pre-treated with either saline (solid bars) or LPS (hashed bars) as neonates. In both treatment groups, we observed an age-related decrease in passive responses, with a parallel increase in more complex responses. However, there were no significant differences in the proportions of the responses to hyperpolarizing current between saline- and LPS-treated, or between the ipsi- and contralateral SDHs at any of the three ages examined.

Injection of hyperpolarizing current revealed one of five responses in SDH neurons (Figure 2, upper panels). Neurons displaying a passive response are characterized by the absence of active conductances during or after the hyperpolarizing current injection. In some neurons, we observed a “sag” in the voltage trace during the hyperpolarization step, which has been associated with the hyperpolarization-activated mixed cationic inward current [I_H_; (29)]. In the remaining neurons, we observed three types of responses upon release from hyperpolarization: an extended hyperpolarization where it took some time (>200 ms) for membrane potential to return to RMP; and a rebound depolarization which occurred with or without AP discharge. These responses upon release from hyperpolarization have been previously associated with calcium subthreshold currents (30).

In all groups, passive responses to hyperpolarizing current injection dominated (Figure 2, lower panels). There were no significant age-related changes in the prevalence of the five responses to hyperpolarizing current injection in either saline- or LPS-treated rats (*G statistic* = 5.92, *p* = 0.31 and *G statistic* = 3.39, *p* = 0.64, respectively). Furthermore, at each age examined, neurons from saline- and LPS-treated rats displayed similar proportions of the responses to hyperpolarizing current injection. There were also no differences between the prevalence of neurons displaying each of the responses to hyperpolarizing current from the ipsi- and contralateral sides of the SDH. As with the AP discharge categories above, it appears that neonatal exposure to LPS does not affect the developmental trajectory of SDH neurons responses to hyperpolarizing current.

### Subthreshold currents

**Figure 3:**
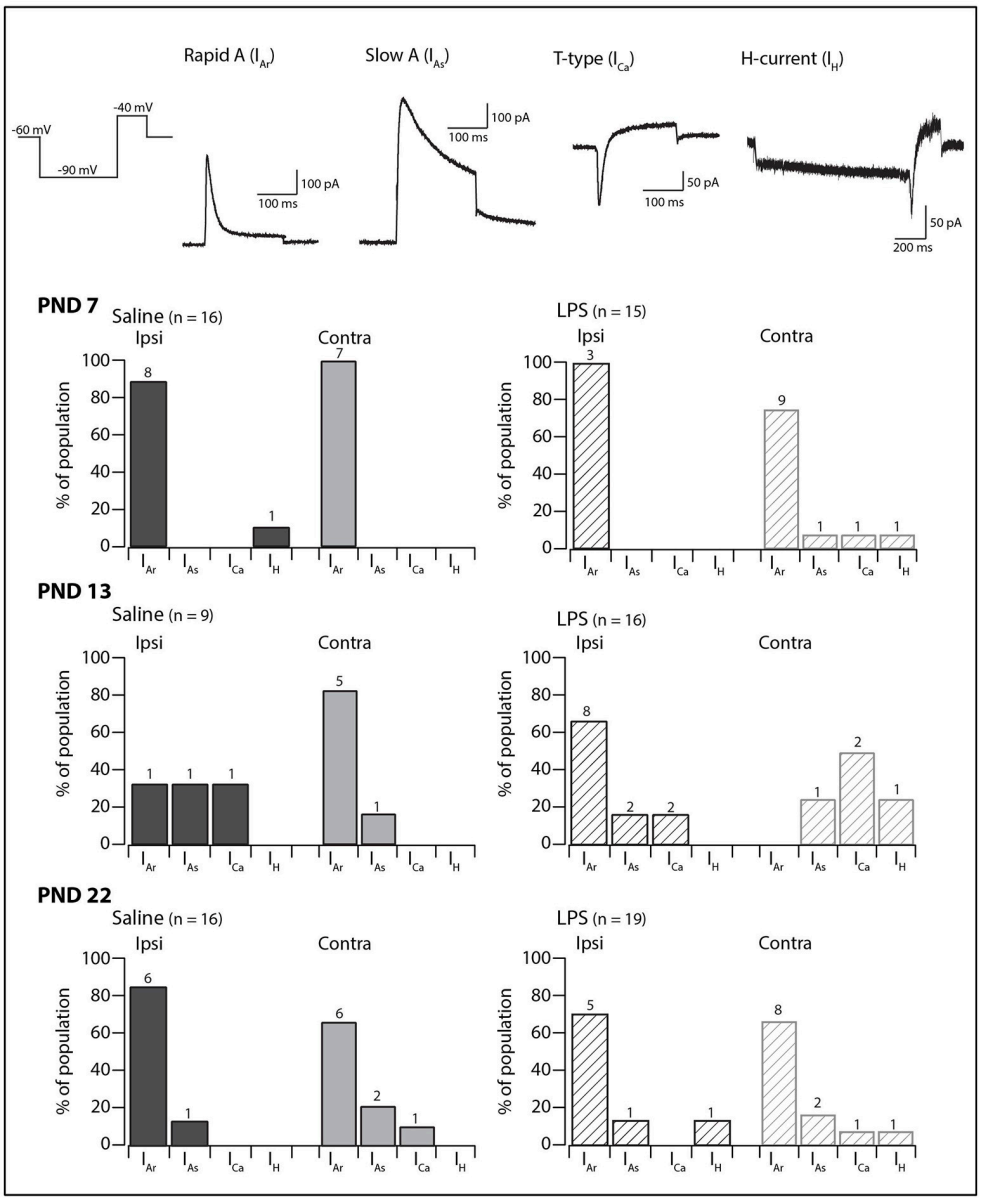
Prevalence of subthreshold currents. Upper traces: representative traces showing the subthreshold currents observed in SDH neurons in response to a two-step voltage protocol (far left). All traces are from saline-treated PND 22 SDH neurons, recorded from a holding potential of −60 mV, with the exception of the H-current (I_H_) which was recorded in an LPS-treated PND 22 SDH neuron. Lower bar plots: demonstrate the prevalence of AP discharge categories at the ages examined (PND 7, 13, and 22, *n* values for each bar indicated). SDH neurons were recorded both ipsilateral (dark gray) and contralateral (light gray) to the formalin injection site in animals pre-treated with either saline (solid bars) or LPS (hashed bars) as neonates. In both treatment groups, an age-related decrease in the rapid-A type current was observed. There were no significant differences in the proportions of the subthreshold current types between saline- and LPS-treated, or between the ipsi- and contralateral SDHs at any of the three ages examined.

We next examined the presence and proportions of subthreshold currents known to underlie the above responses to depolarizing and hyperpolarizing current injection in a subset of SDH neurons (PND 7 = 59; PND 13 = 59; PND 22 = 66). The subthreshold current could not be determined in a subset of these neurons as the currents were obscured by AP discharge during the depolarizing step. In the remaining SDH neurons, one of four major subthreshold currents could be identified: either rapid A (I_Ar_), slow A (I_As_), the T-type calcium current (I_Ca_), or the non-specific cationic current (I_H_) (Figure 3, traces in upper panels). At PND 7 and 22, I_Ar_ was the dominant current on both the ipsi- and contralateral sides of the SDH, regardless of treatment group (saline or LPS). At PND 13, however, the proportion of subthreshold currents was more equitable between I_Ar_, I_As_, and I_Ca_. The non-specific cationic current, I_H_, was rarely observed in any of the groups in our study. A comparison of the proportions of subthreshold currents revealed no developmental differences in either saline- and LPS-treated rats (*G statistic* = 7.26, *p* = 0.20 and *G statistic* = 6.69, *p* = 0.24, respectively). Moreover, there was no difference in the proportions of subthreshold currents between the two treatment groups at any age examined (PND 7: *G statistic* = 4.40, *p* = 0.49; PND 13: *G statistic* = 4.05, *p* = 0.54; and PND 22: *G statistic* = 4.32, *p* = 0.50; Figure 3, bar plots). Therefore, neonatal exposure to LPS has no impact upon the presence and proportions of subthreshold currents in the SDH during development.

### Properties of the rapid A (I_Ar_) current

**Figure 4:**
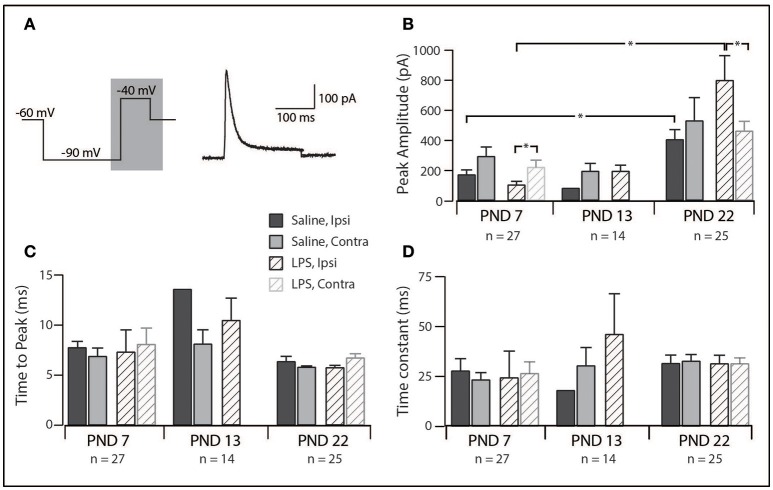
Properties of the rapid-A subthreshold current. **(A)**, Left trace: two-step voltage protocol injected into SDH neurons to examine the presence of any subthreshold currents. Right trace: representative rapid-A current (from a saline-treated PND 22 SDH neuron) observed in response to the protocol on the left. Current trace taken from the area shaded in gray. **(B–D)**, Bar plots demonstrating selected rapid-A current properties for each treatment group, at each age group, both ipsi- and contralateral to the formalin injection. ^*^indicates *p* < 0.05. **(B)**, Peak current amplitude showed an age-related increase and differed between ipsi- and contralateral SDH neurons at PND 7 and PND 22. **(C)**, time taken to reach peak current amplitude remained relatively stable, both between treatment groups and developmentally. **(D)**, decay time constant did not change between ages or treatment groups.

Since I_Ar_ was the dominant current across our sample, we compared its peak amplitude, time to peak and decay time constant across all groups in our study. The distribution of discharge categories from this subset of neurons mirrored the distribution of the overall population (as displayed in Figure 1), therefore, represents an unbiased sampling. A two-way analysis of variance revealed a main effect for the peak amplitude of the I_Ar_ current [*F*_(1, 55)_ = 4.517, *p* = 0.038], with a significant interaction effect between the treatment and spinal cord location [*F*_(1, 55)_ = 4.722, *p* = 0.034]. Developmentally, we observed a significant increase in peak current between PND 7 and PND 22 (Figure 4B; saline ipsilateral —*p* < 0.01; LPS ipsilateral—*p* = 0.02), with no changes in either time to peak or decay time during this developmental period. Furthermore, we observed an increase in I_Ar_ peak amplitude in ipsilateral SDH neurons of the LPS- vs. saline-treated rats (*p* = 0.04). Peak I_Ar_ current amplitude also differed between ipsi- and contralateral SDH neurons in LPS-treated rats at both PND 7 (*p* = 0.04) and PND 22 (*p* = 0.03), with no corresponding changes in either time to peak or decay constant at either age. To account for any possible changes in channel distribution on the somato-dendritic tree of SDH neurons during development, we compared the membrane capacitance in all recorded neurons, and found no difference across any of the groups examined [*F*_(2, 218)_ = 0.008, *p* = 0.992]. Taken together, these data suggest the potassium channels responsible for generating the I_Ar_ current are maturing during the postnatal period between PND 7 and 22, and that this developmental trajectory appears to be susceptible to neonatal LPS exposure.

### Spontaneous excitatory synaptic currents

**Figure 5:**
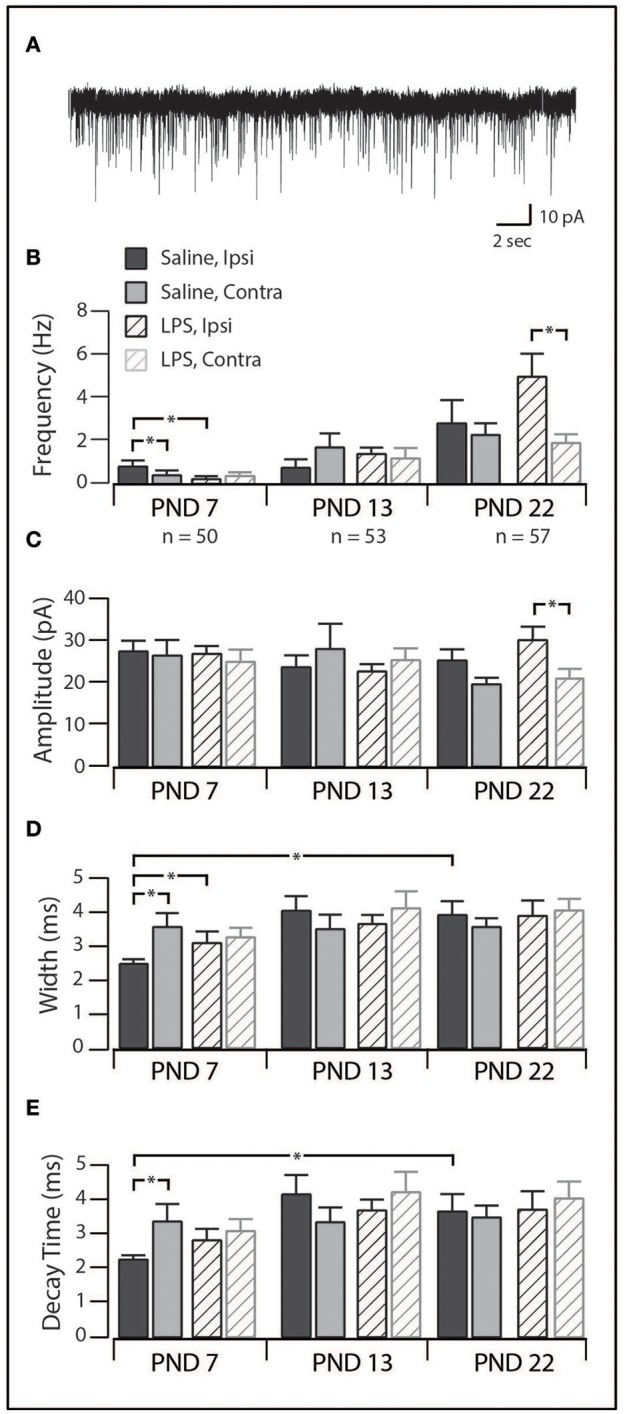
Excitatory synaptic drive in SDH neurons. **(A)**, trace showing representative spontaneous excitatory postsynaptic currents (sEPSCs, 30 s of continuous data). Each downward deflection represents one sEPSC from a saline-treated PND 22 SDH neuron (holding potential −70 mV). **(B–E)**, Bar plots demonstrating selected sEPSC properties for each treatment group, at each age, both ipsi- and contralateral to the formalin injection. ^*^indicates *p* < 0.05. **(B)**, sEPSC frequency displayed an age-related increase, as well as treatment-specific differences at PND 7 and PND 22. **(C)**, sEPSC amplitude remained relatively stable throughout development in both treatment groups. **(D)**, sEPSC width remained relatively stable throughout development in both treatment groups. **(E)**, sEPSC decay time remained relatively stable throughout development in both treatment groups.

In order to assess network excitability in spinal cord slices, we recorded excitatory postsynaptic currents (sEPSCs; holding potential −70 mV) in a subset of SDH neurons (PND 7 = 50; PND 13 = 53; PND 22 = 57). The distribution of discharge categories from this subset of neurons mirrored the distribution of the overall population (as displayed in Figure 1), therefore, represents an unbiased sampling. sEPSCs represent the postsynaptic response to neurotransmitter release (both AP driven and quantal) from presynaptic terminals and are observed as downward deflections on the current traces (Figure 5A). We observed significant alteration of sEPSC parameters [frequency: *F*_(2, 148)_ = 25.84, *p* = 0.00; width: *F*_(2, 140)_ = 4.78, *p* = 0.01; decay: *F*_(2, 140)_ = 4.49, *p* = 0.01] with *post-hoc* comparisons revealing the changes described below. We observed a developmental increase in sEPSC frequency between PND 7 and 22, although this did not reach significance (*p* = 0.06). However, there was a significant increase in sEPSC width (*p* < 0.01) and decay time (*p* < 0.01) between PND 7 and 22. The combination of sEPSC properties produced an sEPSC charge transfer that was similar at all three ages [*F*_(2, 140)_ = 0.563, *p* = 0.571], however, the higher sEPSC frequency at PND 22 indicates an increased excitatory drive accompanies development. Taken together, these data demonstrate that the SDH is a highly plastic network during the first three postnatal weeks, with changes in both the number and location of synaptic connections within the SDH.

When studying the effect of neonatal exposure to LPS, we observed significant changes in the frequency of sEPSCs following formalin injection (Figure 5B). At PND 7, ipsilateral SDH neurons from LPS-treated rats displayed a lower sEPSC frequency (*p* = 0.04) and larger width and decay time (*p* = 0.04) compared to ipsilateral SDH neurons from saline treated rats (*p* = 0.04). Interestingly, in saline treated rats, we also observed an increased sEPSC frequency in ipsilateral compared to contralateral SDH neurons (*p* = 0.01). There were no differences in any sEPSCs parameter observed at PND 13. At PND 22, sEPSCs from LPS-treated rats exhibited a higher frequency (*p* = 0.01) and greater amplitude (*p* = 0.02) in ipsilateral vs. contralateral SDH neurons, however there was no significant differences between rats pre-treated with saline compared to LPS. Since there were no differences in the charge transfer at either PND 7 or 22, these changes in frequency indicate an increase in excitatory drive associated with neonatal LPS treatment. Taken with the developmental changes in the SDH described above, these data suggest neonatal exposure to LPS affects synaptic activity and thus SDH network excitability. Importantly, these changes are expressed upon exposure to a subsequent inflammatory event.

## Discussion

In this study, we examined the electrophysiological properties of SDH neurons in rats exposed to both a bacterial mimetic during the early neonatal period and a later inflammatory challenge at three time points during postnatal development. In addition to examining the long-term effects of neonatal inflammation, we were also able to map the developmental trajectory of SDH neurons in the rat by studying those treated with saline. Overall, we found no changes in the types or proportions of neuronal responses to either depolarizing or hyperpolarizing current. In contrast, we found the amplitude of the rapid A-type potassium increased both developmentally, and in response to neonatal treatment with LPS. We also observed significant changes in SDH network excitability, as measured by sEPSCs properties after a subsequent inflammatory event. Taken together, these data suggest a neonatal immune challenge does not markedly affect the intrinsic properties of SDH neurons, however, it can increase the excitability of local spinal cord networks via altering the properties of their rapid A currents and excitatory synaptic connections. These changes in neurons within spinal cord pain circuits have the capacity to alter nociceptive signaling in the ascending pain pathway.

### Effect of neonatal LPS exposure on SDH neuron properties

We were surprised not to observe any long-term changes in SDH neuron responses to current injection following neonatal LPS exposure. Although our recordings were made several hours following formalin injection, prior studies have demonstrated that formalin injection into the hindpaw can have long-lasting effects. For example, behavioral studies show an increase in thermal and mechanical sensitivity 4 weeks after formalin injection (31) and extracellular recordings of dorsal horn neurons *in vivo* revealing an increase in AP discharge at least 80 min after formalin injection (32). Although studies on the developmental of rat SDH neurons are limited, we have previously identified a “critical period” during mouse SDH neuron development, between PND 6 and PND 10 (11). Furthermore, the HPA axis in rats is known to undergo a “stress hyporesponsive period” (SHRP) from PND 4 to 14 (33), where behavioral responses to the formalin test are blunted (3). Perhaps our LPS challenge, administered at PND 3 and 5, fell within a similar developmental “hyporesponsive period” in the rat SDH, and thus the effect on the responses to current injection was limited. Moreover, it is not known how long the central sensitization resulting from formalin injection persists *in vitro*, especially given the removal of peripheral and descending inputs during the slicing process.

Importantly, most studies on the effect of pro-inflammatory substance levels in the spinal cord have been undertaken in adult animals (8–10) and thus do not consider neuronal plasticity and the differing response of young and adult nervous systems to antigens (34). For example, prostaglandin E2 (PGE2) levels are higher in neonatal versus adult plasma (35). This is interesting as PGE2 is known to modulate cytokines levels (36) and directly depolarize adult dorsal horn neurons when applied to spinal cord slices (6). In addition, whilst peripheral inflammation following formalin injection is known to last up to 4 weeks in the adult hindpaw (31), it is unknown how long this peripheral inflammation persists during the early postnatal period. Together these findings suggest further examination of the neuro-immune interface in the spinal cord at specific stages during development is required to better understand the mechanisms underlying long-term effects of neonatal immune challenges.

The amplitude of the rapid A potassium current increased during development. This suggests the number of channels responsible for generating the rapid A current at PND 22 following neonatal LPS exposure are increased (Figure 4). The rapid A current has been implicated in several developmental disorders (37) and is known to inhibit neuron activity by hyperpolarizing neurons and thus reducing the likelihood of AP discharge (25, 38). Short duration neonatal hypoxia (14–16 min) has been shown to result in a reduction of potassium channel mRNA levels as well as peak current amplitude in hippocampal cells (39). This indicates systemic insults can alter potassium channel expression within central neurons. In our study, the increased amplitude of the rapid-A-type potassium current following neonatal exposure to LPS could be a compensatory mechanism to balance out factors that would increase excitability, such as the increase in sEPSC frequency, within the SDH (38). Several studies have associated the rapid-A-type potassium current with the delayed firing AP discharge phenotype (38). Delayed firing, however, was rarely observed in our sample of neurons. This is best explained by reference to a recent computational study showing that the density and relative ratios of potassium channels can alter the phenotypical AP discharge pattern of SDH neurons (40, 41). Given our findings of altered channel expression during development, it is possible that the critical level of I_Ar_ channels required for delayed firing has not yet been reached and may continue to rise during the subsequent postnatal period.

At PND 22 we observed an increase in sEPSC frequency, width and decay time in SDH neurons from saline-treated rats, with an augmentation of this increase in frequency observed following neonatal exposure to LPS (Figure 5). An increase in sEPSC frequency is thought to indicate increased release probability or an increased number of synaptic connections. One possible factor that determines sEPSC width and decay time is the distance between the location of the synapse and the cell soma (42, 43). Given the increased primary afferent connections within the dorsal horn (44) and increased dendritic-arbor size in SDH neurons during development (45) it is perhaps not surprising that we noted increases in sEPSC width and decay time. Previous studies investigating the long-term impact of neonatal inflammation have demonstrated an augmentation of the developmental increase in the size of the terminal field of the sciatic nerve following injection of carrageenan into the neonatal hindpaw (12). This supports the hypothesis that the properties of the peripheral nervous system can be altered following a neonatal insult. Whilst our model of early-life event involved a systemic, rather than localized, insult, it is also probable that the DRGs of our LPS-challenged rats become “primed” during inflammation within the neonatal period. Indeed, studies in adult animals have shown DRG excitability can increase following a noxious insult (46, 47). Our data suggests exposure to another inflammatory attack (i.e., formalin) can transfer this increased peripheral signaling into the SDH and drive long-term changes in both SDH neuron properties and pain behavior. However, given the potential for corticosterone to alter GABAergic synaptic transmission within the dorsal horn (48), investigation of the inhibitory postsynaptic currents is required to determine the full impact of neonatal LPS exposure on synaptic transmission within the SDH.

### Age-related changes in SDH networks

It is well known that neuronal populations in various regions of the nervous system are highly plastic during early postnatal development (49–51). Surprisingly, limited data exist on the developmental trajectory of the spinal neurons (i.e., those in the SDH) that are crucial for pain signaling. To date, the most comparable study on the excitability of rat SDH neurons reported relatively stable excitability during the first three postnatal weeks (52). The findings in our study, however, show that SDH neuronal responses to current injection (Figures 1, 2) and certain sEPSCs properties (Figure 5) are indeed altered during the first three postnatal weeks. These discrepancies could be attributed to differences in methodologies, with the prior study using perforated patch clamping, compared to our whole-cell recordings. Moreover, the previous study conducted recordings from a pre-determined membrane potential (−60 to −65 mV), whereas all recordings in our study were carried out from the neuron's RMP. Therefore, the reasons for the differences between this previous study and our own remain to be determined.

Importantly, the developmental findings outlined in this study demonstrate similarities with those from other species. For example, murine SDH neurons show changes in the proportion of various types of AP discharge during postnatal development. Most notably, the proportion of tonic firers increases, the proportion of single spikers decrease, and the responses of neurons to hyperpolarizing current becomes more complex during the early postnatal period (11). Moreover, these developmental changes in the responses to current injection are also observed in human SDH neurons (28). Thus, our data suggest the developmental trajectory of rat SDH neurons mimics that of other commonly used laboratory rodents, and, importantly, the development of human SDH neurons. The relevance of animal models to human conditions has been a topic of discussion in recent years (53, 54), with translational failures raising questions about interspecies differences and their relevance, especially in the ascending pain pathway. Given the findings that extreme preterm birth in humans (>26 weeks gestation) is associated with complex, centrally mediated alterations in sensory processing (55) the importance of appropriate laboratory models is critical. The similarities between the developmental trajectory of the rat SDH neurons in this study and those reported previously for both mouse (11) and human (28) suggest our model of neonatal inflammation is appropriate for the investigation of the long-term impact of early life events.

### Long-term implications of LPS exposure in the highly heterogeneous SDH

Our previous studies (3, 4), and others (12, 56, 57), have clearly shown changes occur in the central nervous system in response to a neonatal immune challenge. However, in our study, the changes observed in SDH neurons following neonatal exposure to LPS were subtle. The data presented in Figure 1 through 3 show the SDH is a highly heterogeneous neuronal population with at least five functional types based on AP outputs. It is now well appreciated that, in the adult SDH, these distinct AP discharge categories display a unique suite of ion channels and receptors that not only shape their AP discharge patterns, but also impact their roles within the pain neuroaxis [for review see: (58)]. Whilst in this study we were unable to examine the impact of neonatal LPS exposure on each discharge categories, it is possible that some categories may have been preferentially targeted and thus biased our sample. Indeed, previous work on the long-term effects of BDNF on SDH neurons clearly demonstrated changes within these subpopulations, including a decrease in excitatory drive to tonic firers, but an increase for all other discharge categories, indicating a neuron-specific response (59). Thus, to further elucidate the role of SDH neurons in the altered pain behaviors observed in our model, future studies should target specific subpopulations (60). This would help determine whether any subpopulation is preferentially altered by neonatal exposure to LPS and thus represent a future drug target.

## Conclusions

It is now well accepted that extensive cross-talk exists between the immune and nervous systems, and that early life events can have long-term programming effects on the neuroimmune status (61, 62). We have previously reported developmentally regulated long-term changes in the neural, endocrine, and behavioral responses to inflammatory pain following neonatal LPS exposure (3, 4). The present study builds on these prior findings, by demonstrating subtle, but potentially important, differences in the susceptibility of SDH neurons to noxious inflammatory stimuli later in life. This provides a possible mechanism through which neonatal LPS exposure enhances pain responses later in life.

## Author contributions

MT, IZ, DH, and RC conceived and designed the experiments. MT and IZ conducted the experiments, analyzed the data, and prepared the manuscript. All authors approved the final version of the manuscript.

### Conflict of interest statement

The authors declare that the research was conducted in the absence of any commercial or financial relationships that could be construed as a potential conflict of interest.
